# Flavoromics approach reveals the dynamic changes of non-volatile and volatile compounds in the *Sarcodon imbricatus* soup at different cooking times

**DOI:** 10.3389/fnut.2026.1778654

**Published:** 2026-05-07

**Authors:** Xiang Li, Yiming Tang, Zhongyan Zhu, Chengjian Xu, Nina Yan, Shuangli Xiong, Yu Zhou, Siyu Gu, Jing Xiao, Zheng Zeng, Nan Zhu, Chen-Qiang Wang, Xue-Ming Wang, Hang Xiao

**Affiliations:** 1 School of Food Science and Technology, Dalian Polytechnic University, Dalian, China; 2 College of Culinary and Food Science Engineering, Sichuan Tourism University, Chengdu, China; 3 Xinjiang Guannong Co., Ltd, Korla, China; 4 Department of Food Science, University of Massachusetts, Amherst, MA, United States

**Keywords:** cooking times, HS–GC–IMS, multivariate statistical analysis, relative odor activity value (ROAV), *Sarcodon imbricatus*

## Abstract

This study investigated the effects of different cooking times (30, 60, 90, 120, and 150 min) on volatile and non-volatile compounds in *Sarcodon imbricatus* soup. The *S. imbricatus* soup prepared for 150 min exhibited a remarkably high proportion of umami and sweet free amino acids. The research results show that Asp., Glu, and His are the key taste substances of *S. imbricatus* soup. A total of 37 effective volatile compounds were identified in *S. imbricatus* soup, and five key differential flavor substances were screened out, namely 1-octen-3-one-D, 1-octen-3-ol-D/M, 1-nonanal-D/M, 1-octanal-D, and 1-hexanal-D. It was found, through the relative odor activity values, that the short cooking time (30 min) retained the “mushroom” flavor of *S. imbricatus* soup to the greatest extent. Increasing IMP and AMP may enhance the mushroom flavor of the *S. imbricatus* soup. Based on correlation analysis, when IMP and AMP contents exceed 0.35 mg/kg and 0.28 mg/kg respectively, the relative contents of mushroom-characteristic flavor substances (1-octen-3-one-D and 1-octen-3-ol-D) significantly increase (*p* < 0.05). This research can be applied to flavor optimization and the production process of *S. imbricatus* soup.

## 1 Introduction

*Sarcodon imbricatus*, belonging to the family Bankeraceae and the genus Sarcodon, is a rare and valuable wild edible mushroom. Numerous studies have shown that the polysaccharides and other active substances in *S. imbricatus* possess various health benefits, including anti-fatigue (1), antitumor (2), antioxidant (3), and anticancer (4) properties. In recent years, research on the fungus has mainly focused on its active substances, and research on its flavor is relatively lacking. Wang et al. (5) indicated that there were 37 volatile components in *S. imbricatus*, with aldehydes, alkenes, alcohols, and ketones significantly contributing to its flavor. After enzymatic treatment, the aroma of *S. imbricatus* becomes more pronounced (6).

As a valuable part of China’s cultural heritage, soup has long been appreciated by the public. *S. imbricatus*, known for its high protein content, low fat, abundant carbohydrates, minerals, and bioactive compounds, is used as a key ingredient in soup preparation. The flavor of mushroom soup is influenced by multiple factors. Cooking time is recognized as a key parameter affecting the flavor of mushroom soup, with an appropriate duration balancing the generation and release of flavor compounds to create a more robust and pleasant taste (7). Previous studies have shown that the content of umami substances (GMP + Glu) in shiitake mushroom soup peaks at 60 min of cooking (8), and alcohols and C-8 compounds in matsutake soup gradually degrade with prolonged cooking (9, 10). Therefore, it is deemed crucial to investigate the impact of cooking time on the quality of mushroom soup based on the dynamic changes in volatile flavor compounds.

Although preliminary studies have explored the bioactive components and flavor profiles of edible fungi, systematic research on the dynamic evolution of flavor compounds during the cooking process of *S. imbricatus* remains scarce. This particularly applies to the intrinsic relationship between volatile flavor compounds and non-volatile taste-active substances (such as amino acids and nucleotides). To achieve these objectives, *S. imbricatus* soup was selected as the research object, and an electronic nose combined with headspace gas chromatography-ion mobility spectrometry (HS–GC–IMS) was employed to investigate the impact of cooking time on its volatile flavor profiles. Additionally, orthogonal partial least squares discriminant analysis (OPLS–DA) modeling, heatmap clustering analysis, and relative odor activity value (ROAV) analysis were conducted to preliminarily reveal the differences in volatile flavor compounds among soups cooked for different durations and characterize the flavor changes during the cooking process. Furthermore, quantitative analysis of taste-active substances (e.g., amino acids and nucleotides) and their correlation with flavor substances were integrated into the study.

## 2 Materials and methods

### 2.1 Preparation of soup

The fresh *S. imbricatus* (Yunnan, China) was cut into pellets of uniform size (2 cm × 2 cm). Based on preliminary experiments, the *S. imbricatus* was mixed with water at a ratio of 1:61 (w/v, g/mL). The mixture was cooked in a water bath at 91 °C (Test Instrument Co., Ltd., Tianjin, China) for different cooking times: 30, 60, 90, 120, and 150 min, labeled as A, B, C, D, and E, respectively.

### 2.2 Free amino acid analysis

The free amino acids (FAAs) were extracted and analyzed on an automatic amino acid analyzer (Biochrom Ltd., Cambridge, G. B.) according to Zhao et al. (11). One mL of the sample was combined with 4 mL of 3% (w/v) sulfosalicylic acid and incubated at 0 °C for 1 h. The mixture was then centrifuged at 4 °C at 10,000 × *g* for 15 min. After shaking the supernatant with 2 mL n-hexane, a 2 mL water phase was taken and filtered by a 0.45 μm filter membrane for FAA analysis. The peak area of amino acids was compared with an external standard method to identify and quantify each FAA.

### 2.3 Nucleotide analysis

External standard method was used for quantification. Guanosine 5′-monophosphate (5’-GMP) and Inosine 5′-monophosphate (5’-IMP) were 5 mg each, Adenosine 5′-monophosphate (5’-AMP), Uridine 5′-monophosphate (5’-UMP) and Cytidine 5′-monophosphate (5’-CMP) were 10 mg each (accurate to 0.1 mg). Mixing with ultra-pure water to 100 mL as the nucleotide standard mixture. Chromatographic conditions: Column: Poroshell 120 EC-C18 (4.6 mm × 150 mm × 4 μm); The column temperature was 25 °C, the mobile phase was 0.02 mol/L KH_2_PO_4_^+^ methanol (95:5 *v/v*), the pH was adjusted to 4.5 with 85% phosphoric acid, and the flow rate was 0.6 mL/min. Detector: UV detector, detection wavelength is 254 nm; The injection volume was 10 μL.

### 2.4 Intelligent sensory evaluation

Analysis of *S. imbricatus* soup used an electronic nose (Baosheng Industrial Development Co., Ltd., Shanghai, China). Soup (2.0 mL) was transferred into empty 10 mL vials. The gas was equilibrated in a 50 °C water bath for 15 min and then it was removed (12). We then sampled the headspace gas for analysis. The sensor underwent a cleaning cycle of 120 s, sample preparation took 10 s, and the detection time was 120 s, with a carrier gas flow rate of 300 mL/min. Each sample was tested in five parallel experiments.

### 2.5 HS–GC–IMS analysis

Analysis of *S. imbricatus* soup samples was performed on an HS–GC–IMS device (FlavourSpec^®^, Gesellschaft für Analytische Sensorsysteme GmbH, Dortmund, Germany). A total of 5 mL *S. imbricatus* soup was measured and placed in a 20 mL headspace glass extraction vial, sealing it with a cap. The vial was incubated at 80 °C for 15 min. After incubation, 0.5 mL of the sample was automatically injected into the injector by a heated syringe. The detection conditions were as follows (13): With the aid of nitrogen gas with a purity of 99.999%, the samples were subsequently pushed into an FS-SE-54-CB-1 chromatography column (15 m × 0.53 mm). The nitrogen flow rate program was as follows: initial flow rate of 2.0 mL/min, maintained for 2 min, then increased to 20.0 mL/min over 10 min, further increased to 100 mL/min over the next 10 min, and finally increased to 150 mL/min over the last 10 min. The analytes were eluted into the ionization chamber, and used nitrogen gas with a purity greater than 99.999% as the drift gas at a flow rate of 150 mL/min. Ions were generated in positive ion mode by beta rays (tritium, ^3^H), driven into a 98 mm drift tube, and separated under constant conditions (45 °C; 500 V/cm). Compounds were qualitatively analyzed by comparing retention indices and drift times.

### 2.6 Taste activity value

Taste activity value (TAV) is an indicator to evaluate the contribution degree of taste substance. TAV > 1 indicates that the taste substance has a significant impact on the taste of the sample, and the greater the value, the greater the contribution effect. The calculation formula is as follows:


$$\mathrm{TAV}=\frac{C}{T}$$

Where: C represents the concentration of non-volatile compounds, mg/kg; T represents the taste threshold of a non-volatile compound, mg/kg.

### 2.7 Relative odor activity value analysis

The contribution of each volatile flavor compound to the aroma of *S. imbricatus* soup was evaluated using the ROAV parameter (14). This parameter sets ROAVstan = 100 for the element contributing the most to each sample odor. For other components, the ROAV was computed as follows:


$$\text{ROAV}\approx \frac{C{\%}_{A}}{C{\%}_{\text{Astan}}}\times \frac{T_{\text{stan}}}{T_{A}}\times 100$$

where: C%A represents the relative content of each volatile odor compound (%), TA represents the odor threshold of each volatile odor compound (mg/kg), C%Astan represents the relative content of the component with the highest overall odor contribution in each sample (%), and Tstan represents the odor threshold of the component with the highest overall odor contribution in each sample (mg/kg).

### 2.8 Multivariate statistical analysis

To explore further the differences between *S. imbricatus* soup cooked at different times, multivariate statistical analysis using principal component analysis (PCA) and orthogonal partial least squares discriminant analysis (OPLS–DA) was employed on the HS–GC–IMS data. The relative concentrations of volatile flavor compounds with ROAV ≥ 1 were used as variables in the analysis.

### 2.9 Data analysis

The experimental results are expressed as “mean ± standard deviation.” Data normalization was performed using SPSS 27.0 (IBM Corp., Armonk, NY, USA). The compounds identified HS–GC–IMS were semiquantitatively analyzed by the normalization method, and the relative content of volatile flavor compounds in each sample was calculated.

A radar chart, pie chart, correlation analysis, and relative content chart were generated using Origin 22.0 (Origin Lab Corporation, Northampton, MA, USA). The OPLS-DA plot and the variable importance in projection (VIP) chart were created using SIMCA 14.1 (Umetrics AB, Umea, Sweden). PCA was created at https://www.chiplot.online/, clustering heat map was created at https://www.omicstudio.cn/tool, accessed on August 30, 2024.

## 3 Results and analysis

### 3.1 Analysis of free amino acids

Free amino acids are the main flavor substances in edible fungi. It can be known from Table 1 that a total of 17 free amino acids were detected in the *S. imbricatus* soup, including 7 essential amino acids for the human body, among which umami amino acids accounted for a relatively high proportion. The total amount of free amino acids first decreased and then increased with the extension of the cooking time. During the 30–90 min stage, the amino acid content decreased, which was mainly attributed to the strecker degradation of amino acids and the Maillard reaction during the cooking process, consuming amino acids (15); The content of free amino acids increased during the 90–150 min stage, which might be due to the intense hydrolysis and denaturation of the proteins in the *S. imbricatus* caused by long-term high-temperature cooking (16). Amino acids are classified according to their taste characteristics into umami amino acids, sweet amino acids, bitter amino acids and aromatic amino acids. The content of umami amino acids is: Glu > Ala>Asp>Gly. The content of umami amino acids first increases and then decreases with the increase of boiling time. The content of sweet amino acids is: Arg > Thr > Ser > Pro; The content of bitter amino acids is: Arg > His>Leu > Val > Ile > Cys > Met; The content of aromatic amino acids is: Phe > Tyr.

**Table 1:** Influence of cooking times on free amino acids of *S. imbricatus* soup.

FAAs	Content(mg/kg)					Threshold (mg/kg)	TAV				
	A	B	C	D	E		A	B	C	D	E
Asp	242.34 ± 0.06^b^	226.52 ± 0.22^d^	212.64 ± 0.21^e^	232.73 ± 0.01^c^	257.52 ± 0.10^a^	80	3.029	2.832	2.658	2.909	3.219
Thr*	215.30 ± 0.32^b^	215.75 ± 0.08^b^	214.12 ± 0.02^c^	211.40 ± 0.53^d^	233.52 ± 0.51^a^	2,600	0.083	0.083	0.082	0.081	0.090
Ser	197.50 ± 0.42^b^	192.27 ± 0.14^c^	189.11 ± 0.20^e^	191.11 ± 0.26^d^	212.81 ± 0.01^a^	1,500	0.132	0.128	0.126	0.127	0.142
Glu	1226.62 ± 0.08^b^	1174.41 ± 0.05^d^	1142.34 ± 0.11^e^	1191.36 ± 0.33^c^	1287.13 ± 0.02^a^	110	11.151	10.676	10.385	10.831	11.701
Gly	97.53 ± 0.38^a^	82.81 ± 0.15^e^	83.75 ± 0.14^d^	84.21 ± 0.09^c^	85.14 ± 0.05^b^	2,160	0.037	0.038	0.039	0.039	0.039
Ala	431.53 ± 0.09^d^	446.36 ± 0.15^b^	439.93 ± 0.09^c^	426.52 ± 0.11^e^	462.74 ± 0.08^a^	1,500	0.288	0.298	0.293	0.284	0.308
Cys	33.40 ± 0.07^c^	39.60 ± 0.08^a^	31.95 ± 0.06^d^	34.74 ± 0.04^b^	31.64 ± 0.10^e^	–	–	–	–	–	–
Val*	152.54 ± 0.1^d^	160.51 ± 0.10^b^	153.31 ± 0.01^c^	147.08 ± 0.03^e^	168.51 ± 0.03^a^	400	0.381	0.401	0.383	0.368	0.421
Met*	34.76 ± 0.07^b^	36.23 ± 0.22^a^	32.28 ± 0.04^d^	24.03 ± 0.02^e^	33.81 ± 0.03^c^	300	0.116	0.121	0.108	0.08	0.113
Ile*	98.75 ± 0.10^d^	99.42 ± 0.13^c^	147.21 ± 0.02^a^	99.42 ± 0.01^c^	111.13 ± 0.11^b^	900	0.11	0.11	0.164	0.11	0.123
Leu*	168.65 ± 0.01^c^	181.20 ± 0.01^b^	167.46 ± 0.03^d^	168.62 ± 0.01^c^	189.15 ± 0.04^a^	5,500	0.031	0.033	0.03	0.031	0.034
Tyr	79.32 ± 0.59^e^	89.30 ± 0.08^a^	82.45 ± 0.04^c^	83.52 ± 0.01^b^	79.82 ± 0.02^d^	-	-	-	-	-	-
Phe*	106.54 ± 0.06^c^	107.43 ± 0.06^b^	98.36 ± 0.04^e^	101.82 ± 0.01^d^	111.20 ± 0.02^a^	530	0.201	0.203	0.186	0.192	0.210
His	253.10 ± 0.18^e^	264.10 ± 0.07^d^	273.72 ± 0.07^c^	326.12 ± 0.01^a^	313.87 ± 0.04^b^	200	1.266	1.321	1.369	1.631	1.569
Lys*	137.72 ± 0.01^c^	139.53 ± 0.06^b^	131.53 ± 0.03^d^	128.81 ± 0.03^e^	152.41 ± 0.01^a^	500	0.275	0.279	0.263	0.258	0.305
Arg	469.64 ± 0.05^b^	406.23 ± 0.05^c^	369.12 ± 0.04^e^	378.05 ± 0.24^d^	528.12 ± 0.03^a^		0.939	0.812	0.738	0.756	1.056
Pro	94.37 ± 0.05^c^	95.11 ± 0.09^b^	95.12 ± 0.02^b^	91.21 ± 0.09^d^	99.13 ± 0.02^a^	3,000	0.031	0.032	0.032	0.03	0.033
Total umami FAAs	1998.04 ± 0.42^b^	1930.12 ± 0.18^d^	1878.63 ± 0.06^e^	1934.92 ± 0.12^c^	2092.53 ± 0.09^a^						
Total FAAs	4039.60 ± 1.76^b^	3956.78 ± 0.16^c^	3864.41 ± 0.15^e^	3920.74 ± 0.56^d^	4357.65 ± 0.48^a^						

“*” indicates essential amino acids. A, B, C, D, and E represent different cooking times: 30, 60, 90, 120, and 150 minutes, respectively.

In order to further analyze the contribution of free amino acids to the taste of *S. imbricatus* soup, the taste activity of free amino acids in *S. imbricatus* soup prepared with different cooking times was analyzed. It can be known from Table 1 that among the 17 kinds of free amino acids, the TAV of 11 kinds of free amino acids is ≥0.1. Among them, the TAV values of Asp., Glu and His are all greater than 1, and they are the key flavor-presenting amino acids in *S. imbricatus* soup. The ratio of Ser, Ala, Val, Met, Ile, Phe, Lys and Arg to 0.1 ≤ TAV ≤ 1 has a modifying effect on the taste of *S. imbricatus* soup. The Glu and Asp contribute the most to the taste of *S. imbricatus* soup, and they are the main sources of umami flavor in edible fungi.

### 3.2 Nucleotide analysis

Among the various flavor nucleotides in edible fungi, the main ones are 5’-GMP, 5’-AMP, and 5’-IMP. Among them, 5’-GMP, as the most critical flavor nucleotide component, can synergistically with free amino acids to jointly enhance the umami flavor of edible fungi (17), and is an indispensable freshening substance in edible fungi. As can be seen from Table 2, the content of 5’-GMP in *S. imbricatus* soup generally increases with the extension of cooking time. 5’-GMP has the flavor characteristics of meat and is known as a much stronger flavor enhancer than MSG (18), and the content of 5’-GMP in *S. imbricatus* soup is higher. 5’-GMP can be produced from 5’-XMP by 5’-XMP aminase conversion (19). With the extension of cooking time, the content of 5’-AMP generally decreases first and then flattens out. 5’-AMP has a sweet taste, which can effectively inhibit bitter and astringent tastes (17). According to the above analysis of free amino acids, there are more bitter amino acids in the soup, and 5’-AMP may effectively cover up the bitter taste in the soup. The content of 5’-IMP decreased significantly with the extension of cooking time, which may be because 5’-IMP was easily decomposed into ribosome by heat instability and participated in the Maillard reaction (20).

**Table 2:** Influence of cooking times on nucleotide content of *S. imbricatus* soup.

Nucleotide	Content(mg/kg)				
	A	B	C	D	E
5’-GMP	3.76 ± 0.13^d^	4.26 ± 0.20^c^	5.33 ± 0.26^b^	5.46 ± 0.13^b^	7.81 ± 0.33^a^
5’-AMP	0.35 ± 0.01^a^	0.28 ± 0.00^b^	0.27 ± 0.04^b^	0.29 ± 0.02^b^	0.27 ± 0.02^b^
5’-UMP	0.34 ± 0.02^d^	0.43 ± 0.03^b^	0.49 ± 0.02^a^	0.39 ± 0.01^c^	0.30 ± 0.01^e^
5’-CMP	6.54 ± 0.02^a^	5.35 ± 0.06^c^	5.67 ± 0.01^b^	4.95 ± 0.18^d^	6.42 ± 0.32^a^
5’-IMP	0.56 ± 0.02^a^	0.36 ± 0.01^bc^	0.38 ± 0.02^b^	0.34 ± 0.05^c^	0.18 ± 0.01^d^
Total	11.54 ± 0.18^c^	10.68 ± 0.20^d^	12.13 ± 0.33^b^	11.42 ± 0.11^c^	14.97 ± 0.57^a^

A, B, C, D, and E represent different cooking times: 30, 60, 90, 120, and 150 minutes, respectively.

From the perspective of TAV values alone, the TAV values of nucleotides in *S. imbricatus* soup prepared by cooking for different times and temperatures are all less than 0.1, indicating that the contribution of single nucleotides to the taste of *S. imbricatus* soup is relatively limited. However, the synergy effect of nucleotides and free amino acids in enhancing umami (the umami flavor enhancement coefficient can reach 5–8 times) cannot be ignored and remains the key mechanism for the formation of the umami flavor of *S. imbricatus* soup.

### 3.3 Intelligent sensory evaluation

**Figure 1:**
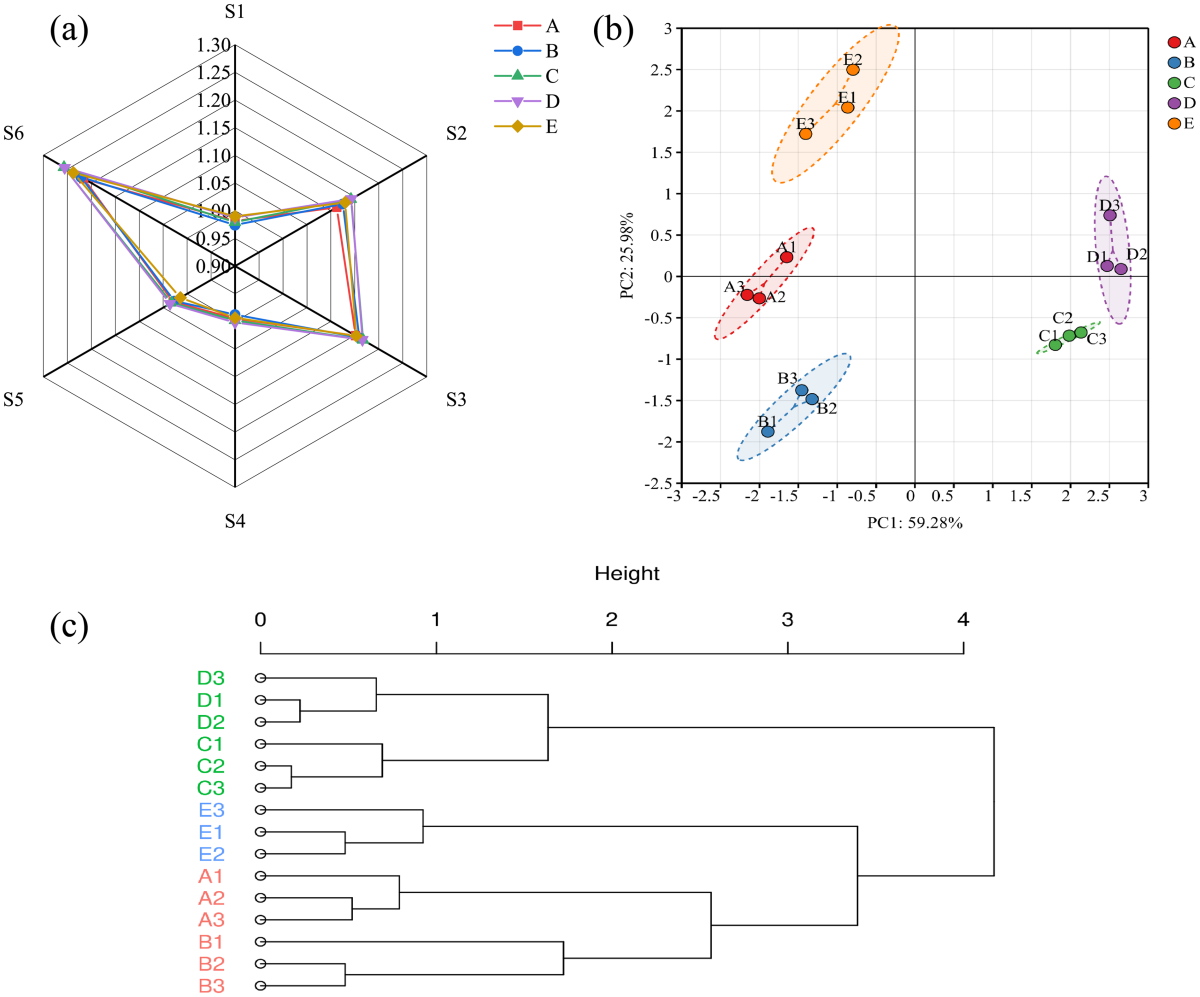
Electronic nose radar **(a)**, PCA **(b)**, and cluster analysis **(c)** of *S. imbricatus* soup at different cooking times.

The electronic nose, which simulates the human olfactory system, analyzes flavor by detecting the volatile compounds in foods (21). To study the overall flavor of *S. imbricatus* soup, an electronic nose was used to analyze the flavor difference in the cooking process of *S. imbricatus* soup. Through electronic nose analysis, it was found that there were differences in the sensor response values of *S. imbricatus* soup with different cooking times (Figure 1a), indicating that the cooking time affected the release mode of volatile components of *S. imbricatus* soup. Among them, the response values of S2 (carbon-containing substances), S3 (hydrogen), and S6 (aldehydes and ketones) sensors were consistently higher than those of other sensors, suggesting that carbon-containing substances, hydrogen and aldehydes and ketones may be generated during the cooking process (the latter may be related to the Maillard reaction). The total response value of the electronic nose increased first and then decreased with the cooking time (the peak value was in group D), indicating that the total volatile components reached the peak when the cooking time was 120 min, and the synchronous change of S6 signal further suggested that the production of flavor substances (such as aldehydes and ketones) might be optimal at this stage.

The effect of cooking time on the electronic nose response value of *S. imbricatus* soup was analyzed by principal component analysis (PCA) which could reduce the dimension and retain the difference of the original data as much as possible. Figure 1b shows that two main dimensions representing data variability were extracted from the PCA of *S. imbricatus* soup, namely, the first principal component (PC1) and the second principal component (PC2), in which PC1 represents the largest part of the differences between samples, and PC2 represents the second largest part of the differences. The component contribution rate represents the proportion of variance explained by that principal component in the data. The cumulative contribution rate of the PC1 and the PC2 in the PCA diagram of *S. imbricatus* soup with different cooking times is 85.3%, which can reflect most of the information of the samples. The five samples can be effectively separated, among which the distance between sample D and sample C is relatively close in the PC1, indicating that some volatiles of samples D and C may be similar. Further cluster analysis (Figure 1c) showed that sample D and sample C could be grouped, and the flavor substances of the two samples were similar. Sample A and sample B were grouped, and the flavor substances of the two samples were similar, which was consistent with PCA.

### 3.4 HS–GC–IMS analysis

#### 3.4.1 HS–GC–IMS spectrum analysis

**Figure 2:**
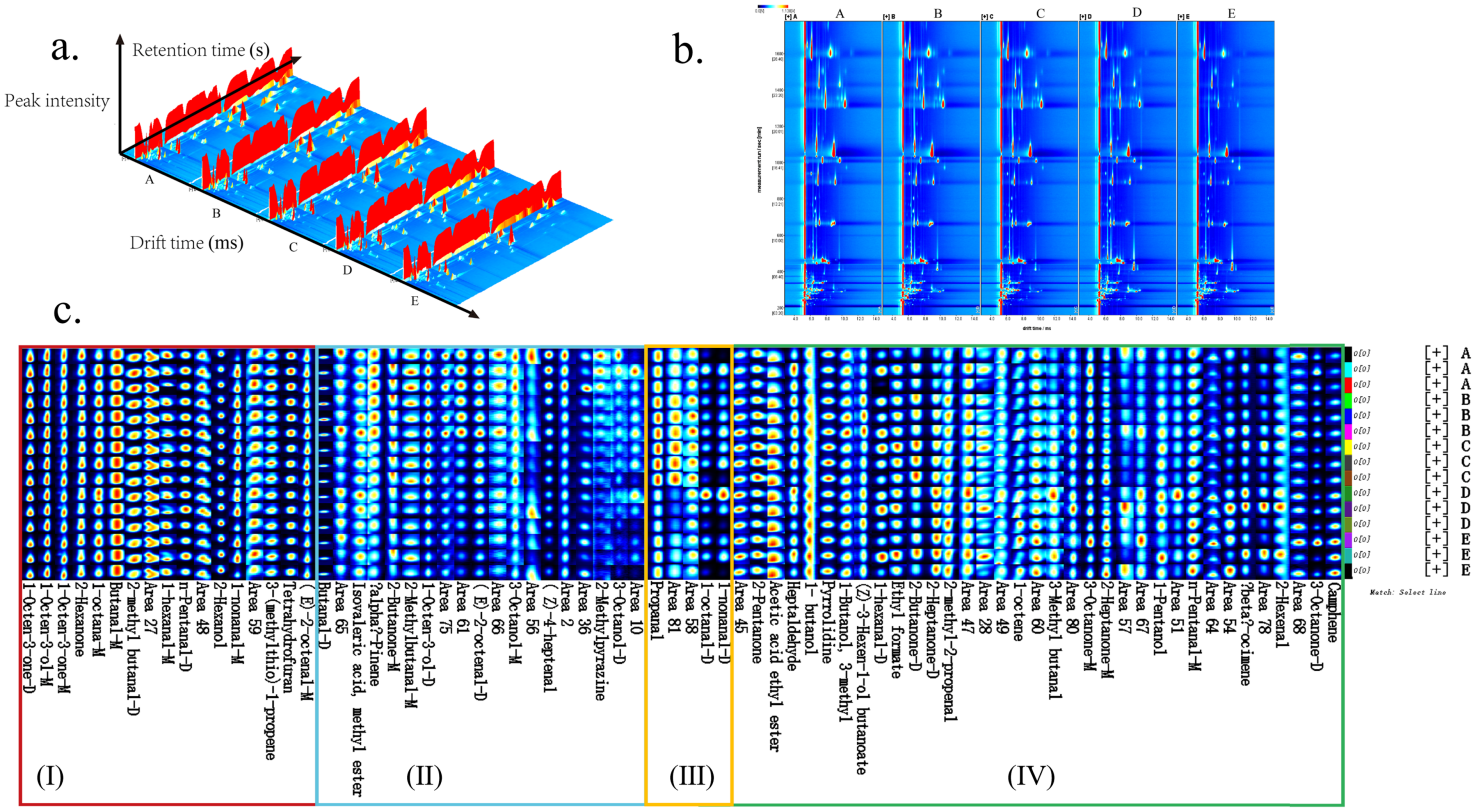
The VOCs in *S. imbricatus* soup with different cooking times based on HS–GC–IMS data. **(A)** Three-dimensional topographic, **(B)** two-dimensional vertical view, **(C)** dynamic fingerprints.

HS–GC–IMS was used to analyze the VOCs of the five *S. imbricatus* soups prepared at different cooking times. Figure 2 shows the two-dimensional and three-dimensional HS–GC–IMS spectra of the *S. imbricatus* soup sample’s distribution of VOCs prepared at different cooking times. According to Figures 2A, B most of the signals of the flavor components of *S. imbricatus* soup with different cooking times were located in the two-dimensional spectral region with a drift time of 4.0–12.0 ms and retention time of 200–1,700 s. The number of signals in the two-dimensional spectrum was almost the same, but the peak signal intensity of each flavor substance was different. The results showed that there was little difference in the flavor components of *S. imbricatus* soup with different cooking times, but the content of the same flavor component was different and had different characteristic spectrum information. In summary, the cooking time affected the volatile flavor substances of the *S. imbricatus* soup.

For instrumental analysis of *S. imbricatus* soup, all the information provided by the fingerprint analysis technique was used for qualitative characterization and was not based on identifying each volatile compound. After chromatographic peak extraction and pretreatment of all flavor component data of the five kinds of *S. imbricatus* soup samples measured using HS–GC–IMS, 50 effective flavor components were obtained, and the NIST database and IMS migration time database built in HS–GC–IMS Library Search software, were used to match. To present the types and concentrations of VOCs among *S. imbricatus* soup samples from different varieties more intuitively, HS–GC–IMS fingerprint spectra were generated using the Gallery Plot plug-in, as displayed in Figure 2C. As shown in Figure 2C, the content of volatile compounds in the *S. imbricatus* soup samples changed greatly with the increase in cooking time. The contents of substances in zone (I) did not change significantly with the extension of cooking time. They mainly included 11 volatile compounds, such as 1-octen-3-one, 1-octene-3-ol-M, 2-hexanone. This showed that these substances were the common flavor substances of the five kinds of *S. imbricatus* soup. The contents of substances in zone (II) decreased gradually with the increase in cooking time, mainly including 7 volatile compounds, such as 1-octene-3-ol-D, (E)-2-octenal-D, alpha-pinene. With the extension of cooking time, the content of substances in zone (III) first increased and then decreased, mainly including 1-nonanal-D and 1-octanal-D. The contents of substances in zone (IV) increased gradually with the extension of cooking time, mainly including 14 volatile compounds, such as ethyl acetate, 3-methyl-1-butanol, (Z)-3-hexen-1-ol butanoate.

#### 3.4.2 Volatile flavor compounds analysis

**Figure 3:**
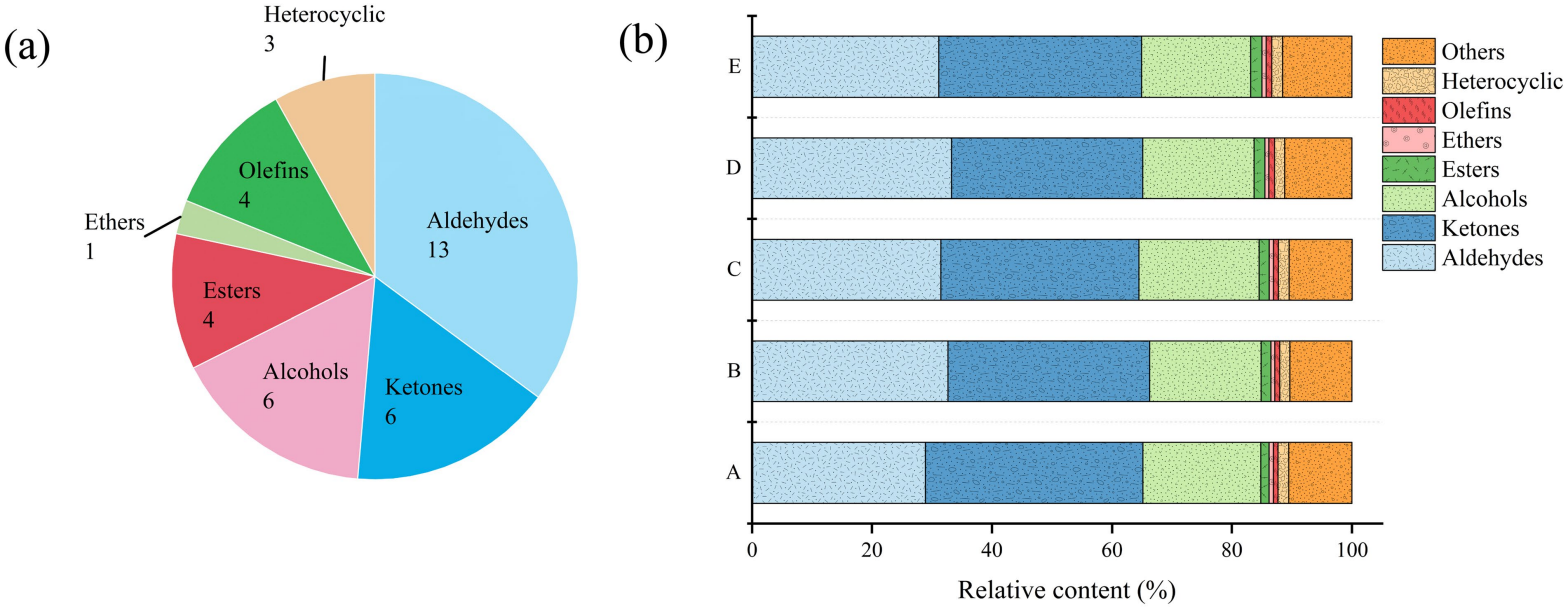
**(a)** The pie charts of the number of VOCs identified using HS–GC–IMS. **(b)** The relative content of each type of VOC determined using HS–GC–IMS.

The relative contents of volatile flavor compounds in each sample are shown in Table 2. Thirteen aldehydes, 6 ketones, 6 alcohols, 4 esters, 1 ether, 4 olefins, and 3 heterocyclic volatile flavor substances were identified in *S. imbricatus* soup samples (Figure 3a), including some dimers of compounds. When the proton affinity of reactants exceed that of high concentration substances in water, protons transfer to substances with higher affinity, thus catalyzing the formation of dimers (22). The HS-GC-IMS detected a total of 50 volatile components (including monomers and dimers), and 37 independent flavor compounds were identified after deduplication.

Figure 3b shows the relative proportion of volatile flavor compounds in the five kinds of *S. imbricatus* soup, which were ketones, aldehydes, alcohols, heterocyclics, esters, olefins, and ethers in order from the largest to the smallest. The relative content of flavor compounds in the *S. imbricatus* soup changed significantly for different cooking times. The relative content of ketones ranged from 31.98 to 36.26%, the largest proportion was 1-octen-3-one, the relative content of aldehydes was 28.92 to 33.26%, mainly including (E)-2-octenal, 1-nonanal, and butanal, and the relative content of alcohols was 18.17 to 20.00%, the main component being 1-octen-3-ol. The relative content of esters was 1.39 to 1.88%, ether was 0.63 to 0.73%, heterocyclics was 1.70 to 1.80%, and the relative content of alkenes was 0.75 to 0.96%. It can be seen that aldehydes, ketones and alcohols are the main volatile flavor substances in *S. imbricatus* soup.

During the preparation of *S. imbricatus* soup, the formation of volatile flavor compounds could be attributed to the Maillard reaction, interactions between amino acids or proteins and oxidized lipids, lipid oxidative degradation, and the degradation of long-chain compounds during heating (23). As shown in Table 3, the content of volatile compounds in *S. imbricatus* soup changed with different cooking times.

**Table 3:** HS–GC–IMS quantitative analysis of *S. imbricatus* soup with different cooking times.

Compound name	Molecular formula	Retention index	Relative content (%)				
			A	B	C	D	E
(*E*)-2-Octenal-*D*	C_8_H_14_O	1432.3	1.30 ± 0.11^a^	1.26 ± 0.08^a^	1.21 ± 0.07^ab^	1.09 ± 0.09^b^	0.88 ± 0.06^c^
(*E*)-2-Octenal-*M*	C_8_H_14_O	1432.6	4.34 ± 0.01^b^	4.18 ± 0.01^c^	4.40 ± 0.01^a^	4.22 ± 0.01^c^	3.97 ± 0.06^d^
(*Z*)-4-Heptenal	C_7_H_12_O	1270.9	0.67 ± 0.05^a^	0.47 ± 0.01^b^	0.38 ± 0.04^c^	0.37 ± 0.04^c^	0.30 ± 0.04^d^
1-Hexanal-*D*	C_6_H_12_O	1081.5	1.08 ± 0.01^d^	1.69 ± 0.01^c^	1.77 ± 0.01^b^	1.94 ± 0.05^a^	1.76 ± 0.03^b^
1-Hexanal-*M*	C_6_H_12_O	1082.3	1.08 ± 0.02^c^	1.21 ± 0.00^b^	1.32 ± 0.03^a^	1.23 ± 0.01^b^	1.33 ± 0.02^a^
1-Nonanal-*D*	C_9_H_18_O	1395.8	2.28 ± 0.01^e^	3.58 ± 0.08^b^	2.99 ± 0.03^c^	3.69 ± 0.07^a^	2.58 ± 0.06^d^
1-Nonanal-*M*	C_9_H_18_O	1396.1	5.04 ± 0.12^c^	6.58 ± 0.09^a^	5.87 ± 0.16^abc^	5.93 ± 0.67^ab^	5.65 ± 0.71^bc^
1-Octanal-*D*	C_8_H_16_O	1292.3	1.30 ± 0.10^bc^	1.43 ± 0.09^b^	1.18 ± 0.10^c^	1.88 ± 0.04^a^	1.30 ± 0.09^bc^
1-Octanal-*M*	C_8_H_16_O	1292.3	2.39 ± 0.09^c^	2.56 ± 0.01^abc^	2.44 ± 0.09^bc^	2.65 ± 0.04^a^	2.62 ± 0.16^ab^
2-Hexenal	C_6_H_10_O	1216.8	0.21 ± 0.01^c^	0.24 ± 0.01^bc^	0.25 ± 0.02^abc^	0.29 ± 0.04^ab^	0.29 ± 0.03^a^
2-Methylbutanal-*D*	C_5_H_10_O	917.8	1.93 ± 0.07^a^	1.79 ± 0.01^c^	1.79 ± 0.01^bc^	1.88 ± 0.06^ab^	1.68 ± 0.06^d^
2-Methyl-2-propenal	C_4_H_6_O	885.6	0.10 ± 0.01^b^	0.12 ± 0.01^ab^	0.13 ± 0.01^ab^	0.13 ± 0.03^ab^	0.15 ± 0.02^a^
2-Methylbutanal-*M*	C_5_H_10_O	909.9	0.17 ± 0.01^a^	0.13 ± 0.01^bc^	0.15 ± 0.01^b^	0.12 ± 0.02^c^	0.14 ± 0.01^b^c
3-Methylbutanal	C_5_H_10_O	881.9	0.08 ± 0.00^c^	0.10 ± 0.00^b^	0.11 ± 0.01^b^	0.14 ± 0.02^a^	0.14 ± 0.01^a^
Butanal-*D*	C_4_H_8_O	837.8	0.11 ± 0.00^ab^	0.11 ± 0.00^a^	0.09 ± 0.00^bc^	0.10 ± 0.00^c^	0.10 ± 0.00^d^
Butanal-*M*	C_4_H_8_O	848.1	3.01 ± 0.28^b^	3.10 ± 0.10^b^	3.45 ± 0.23^ab^	3.48 ± 0.36^ab^	3.85 ± 0.37^a^
Heptanal	C_7_H_14_O	1183.8	1.35 ± 0.01^d^	1.49 ± 0.00^a^	1.33 ± 0.01^e^	1.46 ± 0.01^b^	1.43 ± 0.00^c^
*n*-Pentanal-*D*	C_5_H_10_O	986.2	1.63 ± 0.00^d^	1.76 ± 0.00^b^	1.65 ± 0.04^cd^	1.85 ± 0.04^a^	1.67 ± 0.02^c^
*n*-Pentanal-*M*	C_5_H_10_O	983.5	0.50 ± 0.06^ab^	0.48 ± 0.03^b^	0.53 ± 0.06^ab^	0.53 ± 0.09^ab^	0.63 ± 0.09^a^
Propanal	C_3_H_6_O	782.5	0.35 ± 0.03^b^	0.38 ± 0.01^ab^	0.42 ± 0.03^a^	0.27 ± 0.03^c^	0.37 ± 0.04^b^
Aldehydes (13 in total)			28.92 ± 0.94^b^	32.68 ± 1.79^a^	31.48 ± 1.17^ab^	33.26 ± 1.41^a^	31.13 ± 1.21^ab^
1-Octen-3-one-*D*	C_8_H_14_O	1311.3	20.06 ± 1.89^a^	17.82 ± 0.53^ab^	17.28 ± 0.94^ab^	16.52 ± 1.69^b^	16.23 ± 1.52^b^
1-Octen-3-one-*M*	C_8_H_14_O	1313.6	5.72 ± 0.20^a^	5.59 ± 0.08^ab^	5.72 ± 0.09^a^	5.32 ± 0.27^b^	5.75 ± 0.11^a^
2-Butanone-*D*	C_4_H_8_O	902.4	0.32 ± 0.03^b^	0.39 ± 0.05^b^	0.52 ± 0.06^a^	0.34 ± 0.07^b^	0.42 ± 0.1^ab^
2-Butanone-*M*	C_4_H_8_O	900.5	0.18 ± 0.01^bc^	0.18 ± 0.01^cd^	0.20 ± 0.00^a^	0.17 ± 0.00^d^	0.19 ± 0.00^b^
2-Heptanone-*D*	C_7_H_14_O	1179.9	0.70 ± 0.05^c^	0.95 ± 0.04^b^	1.10 ± 0.09^b^	1.30 ± 0.13^a^	1.43 ± 0.13^a^
2-Heptanone-*M*	C_7_H_14_O	1178.8	1.35 ± 0.03^b^	1.45 ± 0.01^ab^	2.06 ± 0.66^a^	1.70 ± 0.08^ab^	1.93 ± 0.08^ab^
2-Hexanone	C_6_H_12_O	1098.9	1.31 ± 0.04^b^	1.20 ± 0.05^c^	1.31 ± 0.04^b^	1.25 ± 0.00^bc^	1.38 ± 0.02^a^
2-Pentanone	C_5_H_10_O	933.1	0.66 ± 0.11^bc^	0.67 ± 0.03^bc^	0.86 ± 0.07^a^	0.61 ± 0.06^c^	0.76 ± 0.04^ab^
3-Octanone-*D*	C_8_H_16_O	1259.4	2.19 ± 0.10^a^	1.93 ± 0.03^b^	1.35 ± 0.03^c^	1.40 ± 0.01^c^	2.14 ± 0.18^a^
3-Octanone-*M*	C_8_H_16_O	1259.1	3.77 ± 0.03^a^	3.44 ± 0.01^c^	2.97 ± 0.12^d^	3.02 ± 0.07^d^	3.60 ± 0.14^b^
Ketones (6 in total)			36.26 ± 1.92^a^	33.62 ± 2.22^ab^	33.05 ± 0.37^ab^	31.89 ± 1.07^b^	33.82 ± 1.65^ab^
1-Butanol	C_4_H_10_O	1140.5	0.22 ± 0.02^c^	0.27 ± 0.00^bc^	0.27 ± 0.02^ab^	0.29 ± 0.03^ab^	0.31 ± 0.02^a^
3-Methyl-1-butanol	C_5_H_12_O	1206.2	0.47 ± 0.01^c^	0.49 ± 0.00^b^	0.45 ± 0.00^d^	0.50 ± 0.01^b^	0.51 ± 0.00^a^
1-Octen-3-ol-*D*	C_8_H_16_O	1486.6	5.57 ± 0.45^a^	5.11 ± 0.19^ab^	5.54 ± 0.35^a^	4.61 ± 0.44^bc^	4.06 ± 0.35^c^
1-Octen-3-ol-*M*	C_8_H_16_O	1486.9	11.16 ± 0.02^a^	10.59 ± 0.19^c^	11.39 ± 0.09^a^	10.85 ± 0.22^b^	10.89 ± 0.10^b^
1-Pentanol	C_5_H_12_O	1256.9	0.29 ± 0.04^b^	0.34 ± 0.01^ab^	0.36 ± 0.02^ab^	0.42 ± 0.05^a^	0.38 ± 0.11^ab^
2-Hexanol	C_6_H_14_O	1300.2	0.93 ± 0.14^b^	0.95 ± 0.04^b^	1.11 ± 0.12^ab^	1.10 ± 0.18^ab^	1.28 ± 0.16^a^
3-Octanol-*D*	C_8_H_18_O	1406.7	0.33 ± 0.06^a^	0.24 ± 0.02^b^	0.25 ± 0.02^b^	0.24 ± 0.05^b^	0.21 ± 0.03^b^
3-Octanol-*M*	C_8_H_18_O	1407.6	0.63 ± 0.08^a^	0.58 ± 0.01^a^	0.61 ± 0.04^a^	0.54 ± 0.04^a^	0.55 ± 0.03^a^
Alcohols (6 in total)			19.61 ± 0.78^ab^	18.57 ± 0.14^ab^	20.00 ± 1.25^a^	18.55 ± 0.76^ab^	18.17 ± 0.88^b^
(*Z*)-3-Hexen-1-ol butanoate	C_10_H_18_O_2_	1457.3	0.50 ± 0.03^b^	0.65 ± 0.03^a^	0.56 ± 0.06^ab^	0.65 ± 0.06^a^	0.63 ± 0.08^a^
Ethyl acetate	C_4_H_8_O_2_	864	0.35 ± 0.04^d^	0.40 ± 0.01c^d^	0.46 ± 0.02^bc^	0.47 ± 0.05^b^	0.54 ± 0.04^a^
Ethyl formate	C_3_H_6_O_2_	832	0.29 ± 0.04^b^	0.33 ± 0.01^ab^	0.40 ± 0.03^a^	0.38 ± 0.04^ab^	0.39 ± 0.09^ab^
Methyl isovalerate	C_6_H_12_O_2_	1016.1	0.26 ± 0.01^b^	0.26 ± 0.02^b^	0.26 ± 0.02^b^	0.29 ± 0.01^ab^	0.32 ± 0.03^a^
Esters (4 in total)			1.39 ± 0.04^c^	1.64 ± 0.04^b^	1.67 ± 0.09^b^	1.79 ± 0.14a^b^	1.88 ± 0.18^a^
3-(Methylthio)-1-propene	C_4_H_8_S	935.6	0.73 ± 0.08^a^	0.63 ± 0.04^a^	0.73 ± 0.05^a^	0.65 ± 0.06^a^	0.71 ± 0.06^a^
Ethers (1 in total)			0.73 ± 0.08^a^	0.63 ± 0.04^a^	0.73 ± 0.05^a^	0.65 ± 0.06^a^	0.71 ± 0.06^a^
Alpha-pinene	C_10_H_16_	999	0.30 ± 0.02^a^	0.28 ± 0.00^a^	0.24 ± 0.01^b^	0.23 ± 0.01^b^	0.20 ± 0.01^c^
Beta-ocimene	C_10_H_16_	1228.5	0.25 ± 0.05^b^	0.32 ± 0.04^ab^	0.36 ± 0.04^ab^	0.45 ± 0.09^a^	0.42 ± 0.10^a^
1-Octene	C_8_H_16_	834.9	0.12 ± 0.03^ab^	0.12 ± 0.02^ab^	0.11 ± 0.01^b^	0.15 ± 0.01^ab^	0.16 ± 0.03^a^
Camphene	C_10_H_16_	1043.7	0.08 ± 0.01^d^	0.10 ± 0.00^cd^	0.11 ± 0.01^bc^	0.13 ± 0.01^b^	0.16 ± 0.02^a^
Olefins (4 in total)			0.75 ± 0.02^b^	0.81 ± 0.06^b^	0.82 ± 0.04^b^	0.96 ± 0.07^a^	0.94 ± 0.07^a^
2-Methylpyrazine	C_5_H_6_N_2_	1270.3	0.14 ± 0.02^a^	0.10 ± 0.00^b^	0.10 ± 0.01^b^	0.09 ± 0.01^b^	0.10 ± 0.00^b^
Pyrrolidine	C_4_H_9_N	1013.6	0.39 ± 0.05^c^	0.42 ± 0.01^abc^	0.50 ± 0.03^a^	0.41 ± 0.05^bc^	0.49 ± 0.05^ab^
Tetrahydrofuran	C_4_H_8_O	867.4	1.28 ± 0.02^a^	1.20 ± 0.01^b^	1.18 ± 0.01^c^	1.19 ± 0.01^bc^	1.20 ± 0.01^bc^
Heterocyclics (3 in total)			1.80 ± 0.02^a^	1.72 ± 0.06^a^	1.78 ± 0.06^a^	1.70 ± 0.07^a^	1.78 ± 0.02^a^
Others			10.54 ± 0.86^a^	10.33 ± 0.01^a^	10.48 ± 0.29^a^	11.20 ± 0.55^a^	11.56 ± 1.01^a^

M represents the monomer of the compound and D represents the dimer of the compound. A, B, C, D, and E represent different cooking times: 30, 60, 90, 120, and 150 minutes, respectively.

Aldehydes are abundant in *S. imbricatus* soup, with their relative content initially increasing and then decreasing as the cooking time extends, reaching the highest relative content at 120 min. Aldehydes are generally produced through lipid oxidation, Maillard reactions, and amino acid degradation (24). Linear and unsaturated aldehydes, such as 1-hexanal, 1-octanal, n-pentanal, 1-nonanal, and propanal, may be generated from the oxidation of linoleic acid, arachidonic acid, and oleic acid (25). (E)-2-Octenal and (Z)-4-heptenal are unsaturated aldehydes, possibly formed from the oxidation of n-6 polyunsaturated fatty acids (26). The relative content of aldehydes increased with prolonged cooking time, indicating that extended cooking promoted aldehyde formation.

Ketones are mainly produced by the degradation of amino acids and the *β*-oxidation of fatty acids, and they play a role in the Maillard reaction (27). As the cooking time increased, the relative content of ketones decreased. Among them, 1-octen-3-one, an eight-carbon compound, has been shown to form through the continuous catalysis and cleavage of linoleic acid and linolenic acid by lipoxygenase and hydroperoxide lyase (28). As shown in Table 3, the relative content of 1-octen-3-one gradually decreased with extended cooking time, indicating that prolonged cooking depleted 1-octen-3-one.

Alcohols are confirmed to be the main contributors to the “mushroom” flavor in edible fungi (29). Short-chain alcohols are mainly produced by glycolysis, whereas long-chain alcohols are produced by amino acid metabolism (30). Among them, 1-octen-3-ol, also an eight-carbon compound, showed a decrease in relative content with prolonged cooking time, similar to the changes observed in 1-octen-3-one.

Esters are mainly formed through the esterification reaction between fatty acids and alcohols (31). As shown in Table 3, the relative content of esters gradually increased with extended cooking time, reaching the highest value at 150 min. This may be due to the hydrolysis of lipids in the *S. imbricatus* soup, producing unsaturated fatty acids that, upon heating and oxidative degradation, generated a large number of aldehydes, alcohols, and ketones, promoting the esterification reaction involving alcohol compounds.

Alkenes are primarily generated by the auto-oxidation of fatty acids (32). They have a high flavor threshold and do not significantly contribute to flavor formation but may serve as important intermediates in the Maillard reaction, forming heterocyclic compounds that enhance overall flavor. As shown in Table 3, prolonged cooking time promoted the generation of alkenes, thereby enhancing the overall flavor of the *S. imbricatus* soup.

The content of ether compounds in the *S. imbricatus* soup was relatively low. Allyl propyl sulfide, a sulfur-containing compound with a fishy smell, was produced by the degradation of amino acids and originated from the conversion of sulfur-containing amino acids.

Heterocyclic compounds mainly originate from the Maillard reaction. Under heat, heterocyclic amino acids undergo condensation and cyclization reactions, forming pyrrole and pyridine compounds (33). Sugars, amino acids, polyunsaturated fatty acids, vitamin C, and vitamin B1 are important precursors for the formation of furan volatile flavor substances.

#### 3.4.3 Clustering heat map analysis of *S. imbricatus* soup under different cooking time

**Figure 4:**
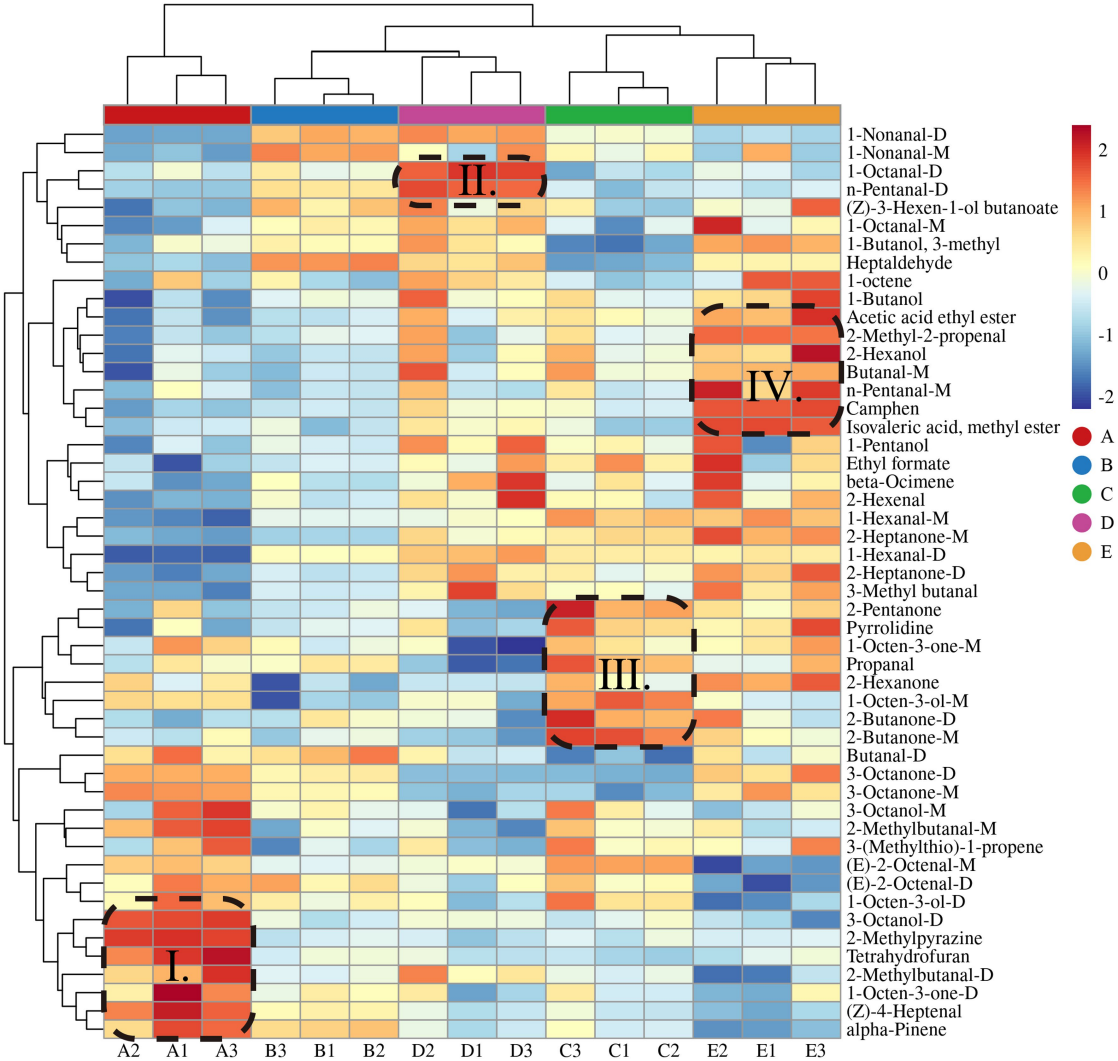
Cluster analysis of heat map of VOCs in *S. imbricatus* soup.

To analyze further the difference of volatile flavor compounds in *S. imbricatus* soup under different cooking times, the relative content of volatile flavor compounds in *S. imbricatus* soup was used as the variable for heat map clustering analysis. As shown in Figure 4, red represents positive correlations, whereas blue represents negative correlations. The five kinds of *S. imbricatus* soup can be significantly clustered into different categories, in which sample A is clustered into a single category, sample B and D are clustered into a single category, and sample C and E are clustered into a single category, indicating that sample B and D are similar in flavor and have significant differences, and sample C and E are also significantly different in flavor similarity. However, this result differs from the electronic nose clustering result, possibly because GC-IMS may detect certain low concentrations but flavor-critical compounds that are not specifically captured in the electronic nose. Electronic nose sensors may respond strongly to certain high-concentration but non-flavor critical substances (such as CO2), masking other signals and only reflecting the overall odor profile. (Z)-4-heptenal, 2-methylbutanal-D, 1-octen-3-one-D, 3-octanol-D, alpha-pinene, 2-methylpyrazine, and tetrahydrofuran were high in sample A (Figure 4I). (Z)-4-heptenal, 1-octen-3-one-D and 3-octanol-D will bring more grassy and mushroom flavor to *S. imbricatus* soup. The contents of 1-octanal-D and n-pentanal-D were higher in sample D (Figure 4II). The two can bring citrus and malty flavor to *S. imbricatus* soup. The contents of propanal, 1-octen-3-one-M, 2-butanone-D/M, 2-hexanone, 2-pentanone, 1-octen-3-ol-M, and pyrrolidine were higher in C samples (Figure 4III). 1-octen-3-one-M and 1-octen-3-ol-M can bring mushroom flavor to *S. imbricatus* soup, but 2-hexanone and 2-pentanone are irritating, which will adversely affect the flavor of *S. imbricatus* soup. The contents of 2-methyl-2-propenal, butanal-M, n-pentanal-M, 2-hexanol, acetic acid ethyl ester, isovaleric acid methyl ester, and camphene were higher in E samples (Figure 4IV). They can bring more fruit and brandy flavor to *S. imbricatus* soup. It can be seen that there are significant differences in the volatile compounds content in the five samples, and these substances give the five samples unique flavor.

### 3.5 Relative odor activity value analysis

The ROAV is used to evaluate the contribution of each flavor compound to the overall flavor of the sample. Because 1-octanal-*M* has a relatively high concentration and a low odor threshold, it contributed the most to the flavor of *S. imbricatus* soup. Therefore, 1-octanal-*M* was set with a ROAV of 100, and the ROAV values of other compounds were calculated accordingly. As shown in Table 4, a total of 17 flavor compounds with ROAV ≥ 0.1 contributed to the flavor of *S. imbricatus* soup during the cooking process. Among them, 11 compounds with ROAV ≥ 1 were key odor components of *S. imbricatus* soup: (*E*)-4-heptenal, 1-hexanal, 1-nonanal, 1-octanal, 1-octen-3-ol, 1-octen-3-one, 2-methylbutanal, butanal-*D*, heptanal, methyl isovalerate, and *n*-pentanal. There are seven flavor compounds with 0.1 ≤ ROAV ≤ 1 that modify the flavor of *S. imbricatus* soup (34): 2-heptanone, 2-hexanone, 2-pentanone, 3-methyl butanal, 3-octanol-*M*, ethyl acetate, and butanal-*M*.

**Table 4:** Odor description and ROAV value of VOCs in *S. imbricatus* soup with different cooking times.

Compound name	Threshold (mg/kg)	Odor characteristics	ROAV				
			A	B	C	D	E
(*Z*)-4-heptenal	0.0034	Grassy-like	5.76	3.81	3.24	2.91	2.34
Alpha-Pinene	1.0138	Pinewood-like	0.01	0.01	0.01	0.01	0.01
1-Butanol	10	Fruity-like	<0.01	<0.01	<0.01	<0.01	<0.01
3-methyl-1-butanol	0.3	Burnt malt-like	0.05	0.04	0.04	0.04	0.05
1-Hexanal-*D*	0.0075	Fresh apple-like	4.21	6.18	6.77	6.85	6.27
1-Hexanal-*M*	0.0075	Fresh apple-like	4.21	4.40	5.07	4.34	4.73
1-Nonanal-*D*	0.015	Fatty-like	4.46	6.51	5.73	6.52	4.61
1-Nonanal-*M*	0.015	Fatty-like	9.86	11.98	11.24	10.46	10.07
1-Octanal-*D*	0.0007	Orange-like	54.46	55.85	48.47	71.16	49.64
1-Octanal-*M*	0.0007	Orange-like	100.00	100.00	100.00	100.00	100.00
1-Octen-3-ol-*D*	0.007	Mushroom-like	23.34	19.96	22.74	17.41	15.50
1-Octen-3-ol-*M*	0.007	Mushroom-like	46.75	41.35	46.74	41.03	41.60
1-Octen-3-one-*D*	0.03	Mushroom-like	19.61	16.23	16.54	14.57	14.47
1-Octen-3-one-*M*	0.03	Mushroom-like	5.59	5.09	5.48	4.93	5.13
1-Pentanol	5	Flowers-like, fruity-like	<0.01	<0.01	<0.01	<0.01	<0.01
2-Butanone-*D*	3	Pleasant fruity-like	<0.01	<0.01	<0.01	<0.01	<0.01
2-Butanone-*M*	3	Pleasant fruity-like	<0.01	<0.01	<0.01	<0.01	<0.01
2-Heptanone-*D*	0.2	Blue cheese-like, nutty-like	0.10	0.13	0.16	0.17	0.19
2-Heptanone-*M*	0.2	Blue cheese-like, nutty-like	0.20	0.20	0.25	0.22	0.26
2-Hexanol	6.7	–	<0.01	<0.01	<0.01	<0.01	<0.01
2-Hexanone	0.09	Pungent alcohol-like	0.43	0.37	0.42	0.37	0.41
2-Methylbutanal-*D*	0.003	Almond-like, malt-like	18.88	16.28	17.14	16.57	17.68
2-Methylbutanal-*M*	0.003	Almond-like, malt-like	1.71	1.20	1.44	1.09	1.23
2-Methylpyrazine	0.25	Hazelnut-like	0.02	0.01	0.01	0.01	0.01
2-Pentanone	0.09	Pungent alcohol-like	0.21	0.20	0.28	0.18	0.23
3-Methylbutanal	0.008	Apple-like	0.30	0.35	0.41	0.45	0.46
3-Octanol-*D*	0.1	Mushroom-like	0.10	0.07	0.07	0.06	0.06
3-Octanol-*M*	0.1	Mushroom-like	0.18	0.16	0.17	0.14	0.15
3-Octanone-*D*	1	Vanilla-like	0.06	0.05	0.04	0.04	0.06
3-Octanone-M	1	Vanilla-like	0.11	0.09	0.09	0.08	0.10
Ethyl acetate	0.1	Brandy-like	0.10	0.11	0.13	0.12	0.14
Butanal-*D*	0.00526	Banana-like	16.80	16.12	18.83	17.52	19.58
Butanal-*M*	0.00526	Banana-like	0.43	0.50	0.58	0.63	0.82
Ethyl formate	6.6	Pungent	<0.01	<0.01	<0.01	<0.01	<0.01
Heptanal	0.01	Fatty-like	3.97	4.08	3.83	3.85	3.83
Methyl Isovalerate	0.0004	Fruity-like	18.92	17.82	18.53	19.47	21.21
*n*-Pentanal-*D*	0.008	Almond-like, malt-like	5.96	6.03	5.92	6.13	5.58
*n*-Pentanal-*M*	0.008	Almond-like, malt-like	1.83	1.63	1.90	1.76	2.10

Aldehyde compounds had a low odor threshold and contributed significantly to the overall flavor of *S. imbricatus* soup, imparting a rich fruity and unique aromatic scent. Medium molecular weight aldehydes (five to nine carbon atoms) had a fresh, fruity, and oily aroma. For example, 1-hexanal has a fresh apple scent, 1-nonanal has a lemon fragrance, and 1-octanal has a citrus aroma (35). In addition, butanal has a banana scent, 2-methylbutanal has an almond and malt aroma, and heptanal has a fatty scent. Ester compounds typically have a sweet fruity aroma (36), a low odor threshold, and can neutralize the off-flavors of most lactones. Methyl isovalerate, with its fruity aroma, contributed to the flavor of *S. imbricatus* soup. Ketone compounds usually have a melon-like and woody aroma, among which 1-octen-3-one and the alcohols 1-octen-3-ol and 3-octanol were eight carbon compounds with a mushroom flavor, being the main contributors to the “mushroom” flavor in edible fungi.

### 3.6 Multivariate statistical analysis

#### 3.6.1 Principal component analysis

**Figure 5:**
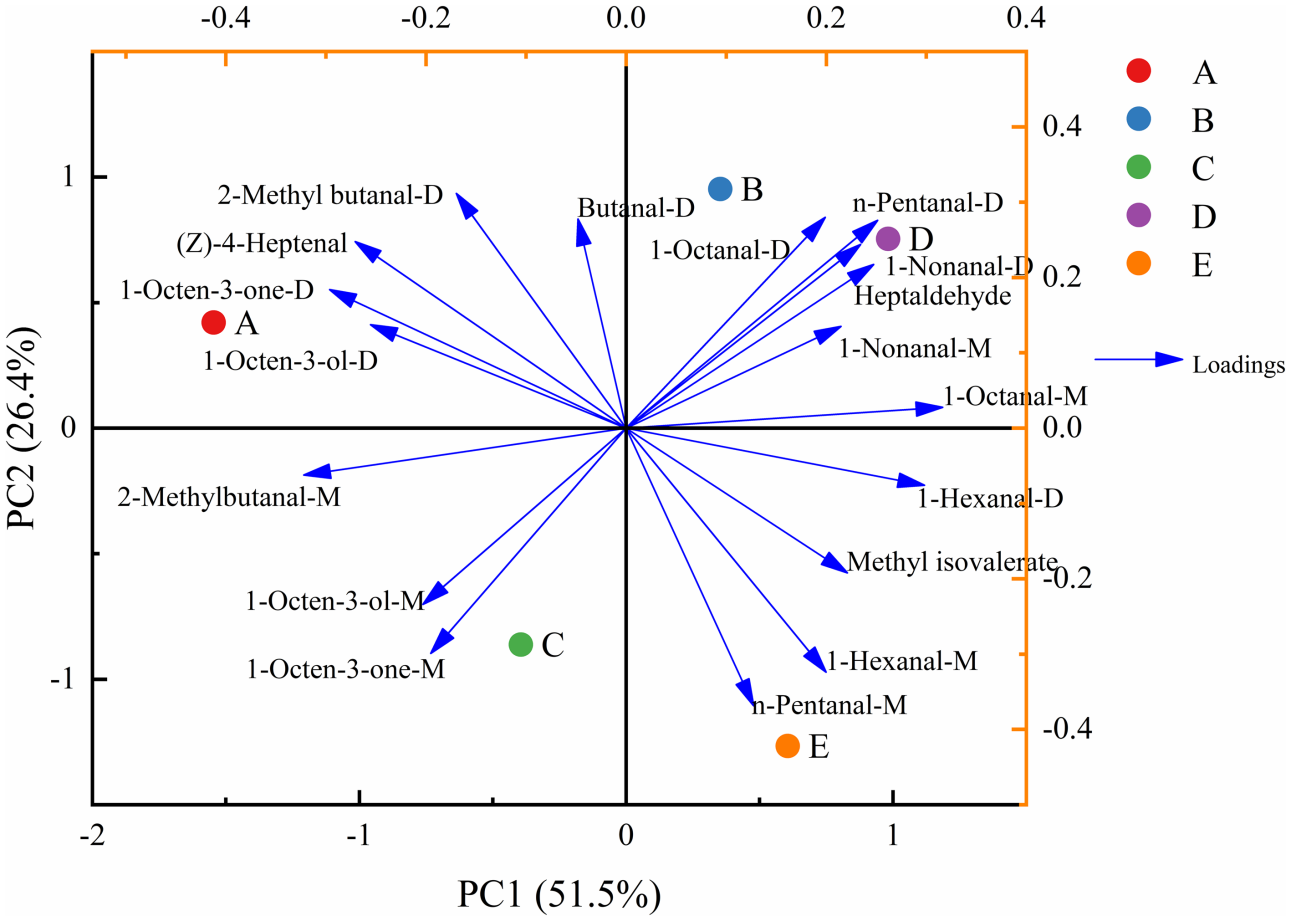
Principal component analysis of VOCs in *S. imbricatus* soup.

For the flavor compounds of *S. imbricatus* soup prepared at different cooking times, unsupervised principal component analysis (PCA) was applied to explore the intrinsic clustering patterns and observe whether a natural separation existed between the groups (37), and the results are shown in Figure 5. The first principal component contributed 51.5% to the variance, while the second principal component contributed 26.4%, totaling 77.9% (<85%). Samples B and D were closely clustered in the same quadrant, indicating similar flavors, the result was similar to the clustering heat map of the whole volatile flavor substances in *S. imbricatus* soup. 1-Octanal-D, n-pentanal-D, 1-nonanal-D, heptanal, and 1-nonanal-M are closely related to B samples and D samples and are the main contributors to the flavor of B and D samples. (Z)-4-Heptenal, 1-octen-3-one-D, 2-methylbutanal-D, and 1-octen-3-ol-D were strongly correlated with A samples. 1-Octen-3-one-M, 1-octen-3-ol-M, and 2-methylbutanal-M were strongly correlated with C samples. n-Pentanal-M, 1-hexanal-M and methyl isovalerate were strongly correlated with E samples. PCA is an unsupervised pattern recognition (PR) classification method, which simply uses variable data for analysis, without considering the output of sample categories, and the information mining between sample categories is insufficient. Therefore, it is necessary to further mine the data information.

#### 3.6.2 Orthogonal partial least squares discriminant analysis

**Figure 6:**
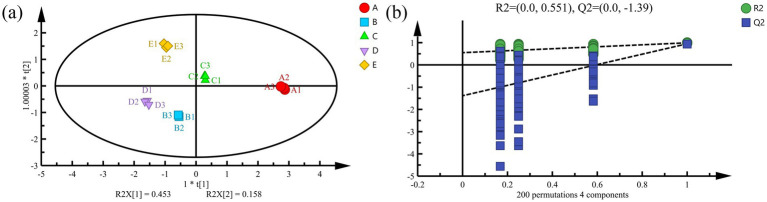
Analysis of VOCs OPLS–DA **(a)** and displacement test **(b)** of *S. imbricatus* soup.

Orthogonal partial least squares discriminant analysis (OPLS-DA), a supervised dimensionality reduction approach, was utilized to maximize the differentiation among predefined sample groups, precisely identify the variables with the highest discriminatory contribution, and minimize the potential for overfitting (37). The OPLS-DA model successfully discriminated among the five *S. imbricatus* soup samples based on their volatile flavor compounds (Figure 6a). The *X* and *Y* matrices explained 0.982 and 0.990 of the variance, respectively, with a *Q*^2^ of 0.933, indicating that the model effectively distinguished between the different cooking times of *S. imbricatus* soup.

The results from the 200-fold cross-validation permutation test are shown in Figure 6b. The plot’s horizontal axis represents the sample retention, with the point at 1 corresponding to the *R*^2^ and *Q*^2^ values of the original model. Both *R*^2^ (0.518) and *Q*^2^ (−1.48) were less than 1, and the *Q*^2^ regression line crossed the negative axis, indicating that the model was reliable and did not suffer from overfitting. This analysis confirmed the robustness of the OPLS-DA model in distinguishing the samples based on their volatile flavor profiles.

### 3.7 Analysis of key differential flavor substances

**Figure 7:**
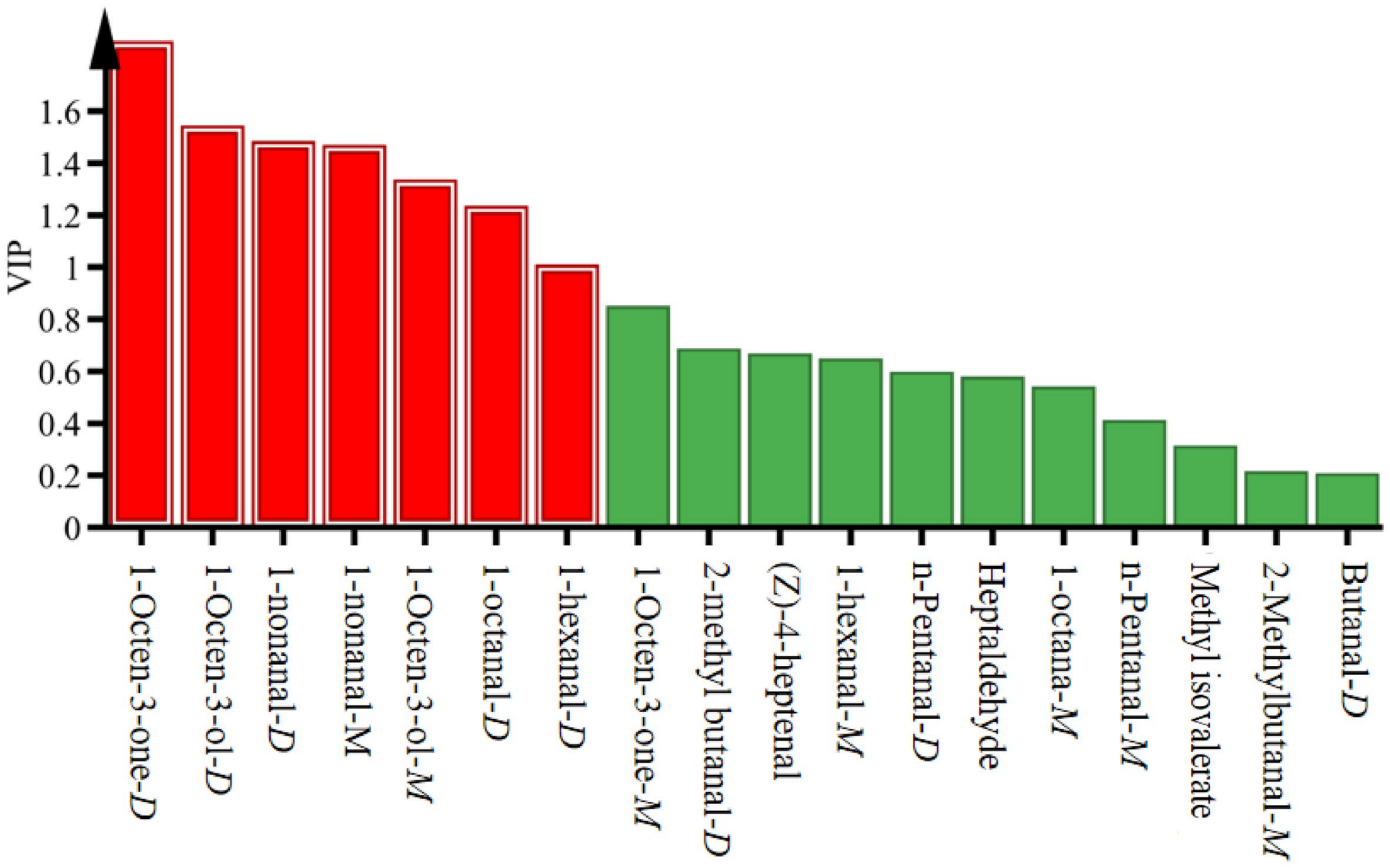
VIP values of VOCs in *S. imbricatus* soup with different cooking times.

The VIP is commonly used to assess the significance of variables in OPLS–DA models, reflecting the contribution of flavor compounds to the classification. A higher VIP value indicates a greater contribution. There were five volatile compounds with VIP value greater than 1, namely 1-octen-3-one-*D*, 1-octen-3-ol, 1-nonanal,1-octanal-*D*, and 1-hexanal-*D* (Figure 7). The VIP value of each flavor substance is listed in Table 5. Therefore, these five volatile flavor compounds were the key different flavor substances in the *S. imbricatus* soup (Includes some dimers and compounds).

**Table 5:** The VOCs VIP value of *S. imbricatus* soup with different cooking times.

Number	Compound name	VIP value
1	1-Octen-3-one-*D*	1.86954
2	1-Octen-3-ol-*D*	1.54485
3	1-Nonanal-*D*	1.48461
4	1-Nonanal-*M*	1.47006
5	1-Octen-3-ol-*M*	1.33711
6	1-Octanal-*D*	1.23644
7	1-Hexanal-*D*	1.01005

These key differentiating compounds belonged to the common C_6_–C_9_ compounds in edible fungi, which were mainly generated by the oxidation of fatty acids. The 1-Octen-3-one-*D* was the substance with the largest VIP value among the five compounds screened, and it was the substance that contributed the most to the flavor difference of the samples in the soup with different cooking times, indicating that the cooking time had the greatest influence on the mushroom flavor in *S. imbricatus* soup (Table 5). As can be seen from Tables 4, 5, 1-octen-3-one-*D* and 1-octen-3-ol also had mushroom flavor, and their ROAV values and relative contents were the largest when the cooking time was 30 min. Therefore, 1-octen-3-one and 1-octen-3-ol were fully released or generated in *S. imbricatus* soup with a cooking time of 30 min. At this time, they contributed the most to the flavor of the mushroom soup, and the mushroom flavor of *S. imbricatus* soup was the strongest. 1-Octanal-*D* has a citrus flavor, 1-hexanal-*D* has a fresh apple flavor. The ROAV value and relative content of both of them were the largest when the cooking time was 120 min, which was conducive to the release or formation of 1-octanal-*D* and 1-hexanal-*D*. When the cooking time was 120 min, the citrus and apple flavor of *S. imbricatus* soup was the strongest. 1-Nonanal-*D* and 1-nonanal-*M* had fat flavor because when the affinity of protons is large, the protons transferred to 1-nonanal-*M* with higher affinity, thus catalyzing the formation of dimer 1-nonanal-*D* (22). They were essentially the same substance. Therefore, when the two were combined and analyzed, their relative content and ROAV value were the largest when the cooking time was 60 min. The cooking time of 60 min was conducive to the formation and release of 1-nonanal, the contribution of 1-nonanal to the flavor was the greatest, and the oil flavor of the soup was more intense. To sum-up, *S. imbricatus* soup had the strongest mushroom flavor when the cooking time was 30 min, and the short cooking time retained the mushroom flavor of the soup to the greatest extent. When the cooking time was 60 min, the fat flavor was the strongest. When the cooking time was 120 min, the citrus and apple flavors were the strongest.

### 3.8 Correlation analysis

Pearson correlation analysis was conducted on the key differential substances and key free amino acids and nucleotides of the *S. imbricatus* soup.

**Figure 8:**
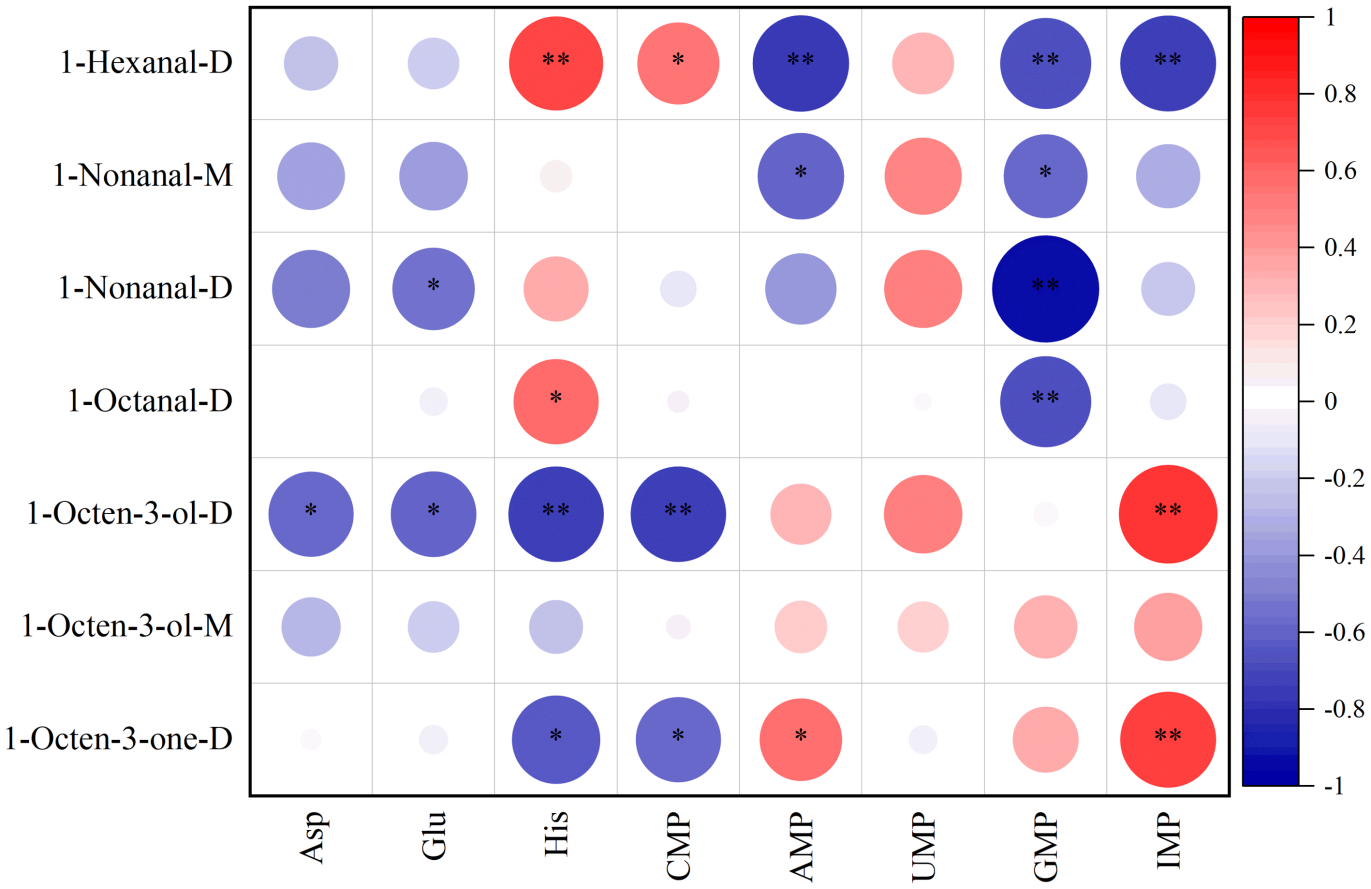
Correlation analysis was performed on the key differential substances, key free amino acids, and nucleotides in the *S. imbricatus* soup.

Figure 8 shows that Asp is significantly negatively correlated with 1-octen-3-ol-D. Asp is an intermediate in the TCA cycle and may inhibit lipid oxidation by competing for carbon sources, thereby reducing the production of 1-octen-3-ol-D (mushroom flavor) (38). Clu was significantly negatively correlated with 1-octen-3-ol-D and 1-nonanal-D. Glutamic acid is involved in nitrogen metabolism and may reduce the accumulation of aldehydes (1-nonanal-D) by inhibiting the activity of aldehyde dehydrogenase or promoting the conversion of aldehydes to acids. His was significantly negatively correlated with 1-octen-3-one-D and extremely negatively correlated with 1-octen-3-ol-D. His was negatively correlated with ketones (1-octen-3-one-D), which might reduce the conversion of ketones to alcohols by inhibiting the activity of ketone reductase. CMP was significantly negatively correlated with 1-octen-3-one-D and extremely significantly negatively correlated with 1-octen-3-ol-D, respectively. CMP may reduce the accumulation of 1-octen-3-one-D and 1-octen-3-ol-D by inhibiting the LOX pathway or promoting the degradation of ketone or alcohol volatiles (39). AMP was significantly and extremely significantly negatively correlated with 1-nonanal-D and 1-hexanal-M, respectively. AMP may inhibit aldehyde-generating enzymes, resulting in a reduction of 1-nonanal-D and 1-hexanal-M (40). GMP was significantly negatively correlated with 1-nonanal-D, 1-octanal-D and 1-hexanal-M, and significantly negatively correlated with 1-nonanal-D. CMP may act as a substrate to compete for phosphate groups and interfere with ATP-dependent enzyme activities (41). GMP may inhibit the accumulation of C6-C9 aldehydes (1-hexanal, 1-octanal, 1-nonanal) by activating aldehyde dehydrogenase or promoting the conversion of aldehydes into other products (42). IMP is extremely significantly negatively correlated with 1-hexanal-M. IMP may inhibit aldehyde dehydrogenase and reduce the accumulation of aldehydes (1-hexanal) (43).

His was significantly positively correlated with 1-octanal-D and extremely significantly positively correlated with 1-nonanal-D. His can act as a histamine precursor. Histamine may activate lipoxygenase (LOX) and promote the generation of C8-C9 aldehydes (1-octanal-D, 1-nonanal-D). AMP was significantly positively correlated with 1-octen-3-one-D. As an energy signaling molecule, AMP may activate the lipid oxidation pathway and promote ketone generation (44). IMP was highly significantly positively correlated with 1-octen-3-one-D and 1-octen-3-ol-D. IMP is a core product of purine metabolism and may promote the oxidation of linoleic acid to 1-octen-3-one and 1-octen-3-ol (mushroom flavor) by activating the LOX pathway or hydroperoxide lyase.

Comprehensive analysis reveals that increasing IMP and AMP may enhance the mushroom flavor (1-Octen-3-one-D, 1-Octen-3-ol-D) of the *S. imbricatus* soup, while inhibiting the grassy flavor (1-hexanal). Adding His or reducing GMP can enhance C8-C9 aldehydes (fruity and fatty aroma).

## 4 Conclusion

In this study, electronic nose and GC-IMS were used to analyze the variation and difference of the flavor of *S. imbricatus* soup under different cooking times. Asp., Glu, and His are the key taste substances of *S. imbricatus* soup. Thirty-seven kinds of volatile compounds were detected in 5 kinds of soup with different cooking times. Among them, aldehydes, ketones and alcohols are the main flavor components in *S. imbricatus* soup. OPLS-DA further indicated that 5 kinds of *S. imbricatus* soup could be effectively distinguished, and 4 key flavor substances were screened out by variable important projection factors: 1-octen-3-one-*D*, 1-octen-3-ol, 1-nonanal*-D/M*, 1-octanal-*D*, and 1-hexanal-*D*. Combined with ROAV analysis, it was found that short cooking time can maximize the retention of *S. imbricatus* soup mushroom taste. Increasing IMP and AMP may enhance the mushroom flavor of the *S. imbricatus* soup. Adding His or reducing GMP can enhance fruity and fatty aroma. On view of the limitations of GC–IMS technology, it is necessary to further study with GC–O (gas chromatography-olfactory measurement) and GC–MS (gas chromatography–mass spectrometer) and other analytical techniques to explore the generation of flavor substances in the cooking process of *S. imbricatus*, so as to provide objective basis for high-quality cooking of *S. imbricatus*. The research findings provide technical guidance for regulating the quality of *S. imbricatus* soup and offer data support for studying the flavor characteristics of *S. imbricatus*.

## Data Availability

The original contributions presented in the study are included in the article/supplementary material, further inquiries can be directed to the corresponding author/s.
